# Early-Life Skin Microbial Biomarkers for Eczema Phenotypes in Chinese Toddlers

**DOI:** 10.3390/pathogens12050697

**Published:** 2023-05-11

**Authors:** Yehao Chen, Yuping Song, Zigui Chen, Jennifer Wing Ki Yau, Kate Ching Ching Chan, Agnes Sze Yin Leung, Oi Man Chan, Apple Chung Man Yeung, Connie Lai Yuk Yuen, Paul Kay Sheung Chan, Wing Hung Tam, Ting Fan Leung

**Affiliations:** 1 Department of Paediatrics, The Chinese University of Hong Kong, Prince of Wales Hospital, Shatin, Hong Kong SAR 999077, China; yehao.chen@link.cuhk.edu.hk (Y.C.); ypsong@link.cuhk.edu.hk (Y.S.); wingki_yau@link.cuhk.edu.hk (J.W.K.Y.); katechan@cuhk.edu.hk (K.C.C.C.); agnes.syl@cuhk.edu.hk (A.S.Y.L.); oimanchan@cuhk.edu.hk (O.M.C.); 2 Department of Microbiology, The Chinese University of Hong Kong, Prince of Wales Hospital, Shatin, Hong Kong SAR 999077, China; zigui.chen@cuhk.edu.hk (Z.C.); appleyeung@cuhk.edu.hk (A.C.M.Y.); paulkschan@cuhk.edu.hk (P.K.S.C.); 3 Hong Kong Hub of Paediatric Excellence, The Chinese University of Hong Kong, Shatin, Hong Kong SAR 999077, China; 4 Department of Obstetrics and Gynaecology, The Chinese University of Hong Kong, Prince of Wales Hospital, Shatin, Hong Kong SAR 999077, Chinatamwh@cuhk.edu.hk (W.H.T.)

**Keywords:** biomarker, birth cohort, eczema, microbiome, skin

## Abstract

Eczema is a common inflammatory skin disorder during infancy. Evidence has shown that skin-microbiome fluctuations may precede eczema development, but their predictive value for eczema phenotypes remains unknown. We aimed to investigate the early-life evolution of the skin microbiome and its temporal associations with different pairs of eczema phenotypes (transient versus persistent, atopic versus non-atopic) in Chinese children. We followed 119 term Chinese infants from birth to 24 months old within a Hong Kong birth cohort. The skin microbes at the left antecubital fossa were serially sampled by flocked swabs at 1, 6, and 12 months for bacterial 16S rRNA gene sequencing. The atopic sensitization at 12 months was strongly associated with eczema persisting to 24 months (odds ratio 4.95, 95% confidence interval 1.29–19.01). Compared with those with non-atopic eczema, the children with atopic eczema had reduced alpha diversity at 12 months (*p* < 0.001) and transiently higher abundance of the genus *Janibacter* at 6 months (*p* < 0.001). Our findings suggest that atopic sensitization at 12 months may predict persistent eczema by 24 months, and atopic eczema at 12 months is associated with unique skin microbiome profiles at 6 and 12 months. Non-invasive skin-microbiome profiling may have predictive value for atopic eczema.

## 1. Introduction

Eczema is a common pruritic, inflammatory skin disorder among children. It affects up to 20% of children in most countries worldwide [1], and this prevalence has increased by two- to three-fold in the past few decades in industrialized countries [2]. Various cohort studies identified different eczema phenotypes based on the disease’s course [3,4,5]. Eczema was also classified into atopic and non-atopic forms, depending on the IgE-sensitization status against inhalant and/or food allergens in the blood, or by skin-prick test (SPT) [6]. Identifying potential early-life biomarkers predictive of these phenotypes may improve our understanding of the pathobiological mechanisms underlying various observable characteristics. It may also contribute to the development of personalized management strategies for childhood eczema at an early age.

Eczematous patients were associated with reduced microbial diversity and the predominance of *Staphylococcus aureus* colonization [7]. Two longitudinal studies further reported that a dysregulated skin microbiome preceded eczema onset. Kennedy et al. used bacterial 16S rRNA gene sequencing to analyze longitudinal skin-microbiome samples in an Irish birth cohort, and reported that coagulase-negative staphylococci colonization at two months of age might predict a lower risk of eczema by one year of age [8]. Meylan et al., using a culture-based study design, reported that colonization by *Staphylococcus aureus* occurred two months before the development of eczema [9]. Nevertheless, there is limited evidence of the role of the early-life evolution of skin-microbiome eczema susceptibility and its phenotypes. This study hypothesized that pediatric eczema patients would possess distinct skin-microbiome profiles for different disease phenotypes, and that changes in the skin microbiome would occur prior to the development of specific eczema phenotypes. This birth-cohort study characterized the early-life evolution of the skin microbiome in Chinese children and examined its temporal associations with different phenotypes of childhood eczema.

## 2. Materials and Methods

### 2.1. Study Subjects

The Stool Microbiome and Allergic ReacTion (SMART Baby) was an observational birth-cohort study conducted in Hong Kong. This study primarily investigated the associations between allergy development and early-life factors, particularly microbial biomarkers. The SMART Baby study recruited Chinese pregnant mothers at gestational age ≥ 37 weeks from the antenatal wards at our university-affiliated teaching hospital between September 2017 and March 2018. Enrolled mothers provided informed written consent. The Chinese University of Hong Kong-New Territories East Cluster Joint Clinical Research Ethics Committee approved this study.

We followed the newborns throughout their first 24 months of life, from September 2017 to June 2020. The skin health and allergy risks of all infants were assessed at 6, 12, and 24 months of age. At 6-month and 12-month clinic visits, pediatricians assessed children’s skin condition and made the diagnosis of eczema according to the Hanifin and Rajka criteria [10]. At 12 months, children underwent SPT with locally important foods, including cow’s milk, hen eggs, soya bean, peanut, wheat (ALK-Abelló, Round Rock, TX, USA), and mixed fish (Greer Laboratories, Boston, MA, USA), as well as *Dermatophagoides pteronyssinus* [11,12,13]. Histamine (10 mg/mL) and normal saline were included as positive and negative controls, respectively. Due to the COVID-19 pandemic, eczema status at 24 months was reported by parental questionnaires instead of being assessed by pediatricians in the clinic.

### 2.2. Definition of Eczema Phenotypes

Subjects who were not diagnosed with eczema during their first two years of life were classified into the ‘never eczema’ group. The other subjects were classified according to the duration of their eczema. Subjects who had eczema by 6 months but went into remission by 24 months were categorized as ‘early-onset transient eczema’. Those who still had active eczema at 24 months were categorized as ‘early-onset persistent eczema’. Eczematous subjects were also classified into ‘atopic eczema’ and ‘non-atopic eczema’ phenotypes based on their atopy status at 12 months. Atopy was defined as at least one positive reaction to allergens by SPT. Reactions were considered positive when average wheal diameter was ≥3 mm larger than that of the negative control.

### 2.3. Sampling for Microbiome Analysis

Skin microbial samples were longitudinally collected at 1, 6, and 12 months. Briefly, subjects were instructed not to bathe or apply any topical cream within 8 h before sampling. At each visit, sterile FLOQSwabs^®^ (COPAN Diagnostics, Murrieta, CA, USA) moistened with autoclaved ST solution (0.15 mM NaCl, 0.1% Tween 20) were used to swab a 4 cm × 4 cm area over left antecubital fossa for one minute. The antecubital fossa was chosen due to its common involvement in eczema. After cutting off the swab shaft with sterile scissors, swab tips were stored at −80 °C in a sterile 1.5 mL centrifuge tube until processing.

### 2.4. Characterization of Skin Microbiome

Bacterial DNA was extracted from skin swab samples using the PureLink™ Microbiome DNA Purification Kit (Invitrogen, Waltham, MA, USA). A 27F/534R primer pair (27F: 5′-AGAGTTTGATCCTGGCTCAG-3′; 534R: 5′-ATTACCGCGGCTGCTGG-3′) was applied with bacterial 16S rRNA DNA (V1–V3 hypervariable regions) amplification. The purified libraries of amplified 16S gene regions were sequenced on Illumina MiSeq PE300 by the Core Utilities for Cancer Genomics and Pathobiology at our University.

The paired-end demultiplexed raw fastq sequences were merged using FLASH (fast length adjustment of SHort reads) [14] to form single long reads. These reads were then imported into QIIME2 for downstream analysis, in which DADA2 [15] was used to denoise the reads and cluster them into amplicon sequencing variants (ASVs). The SILVA v132 99% gene-reference database was used in bacterial taxonomy assignment. Taxa present less than three times in ≥20% of samples were removed from the analysis (https://joey711.github.io/phyloseq/preprocess.html, accessed on 16 April 2022) [16]. More detailed methods are outlined in the Supplementary Materials section of this article.

### 2.5. Statistical Analysis 

The *phyloseq* R package [17] was used for microbiome analysis. Differences in alpha diversity across time, as represented by Shannon and Simpson indices, were tested by Friedmann test with post hoc Nemenyi tests using *PMCMRPlus* R package [18]. Alpha diversities among different eczema phenotypes were compared using the Wilcoxon rank-sum test. The effects of time and eczema phenotypes on beta diversity, as assessed by unweighted UniFrac distance metric, were tested by permutational multivariate ANOVA (PERMANOVA) using *adonis* function in the *vegan* R package [19]. Differentially abundant taxa across time and among phenotype groups were analyzed by analysis of compositions of microbiomes with bias correction (ANCOM-BC) method using *ANCOMBC* R package [20].

## 3. Results

### 3.1. Characteristics of Study Population 

**Figure 1:**
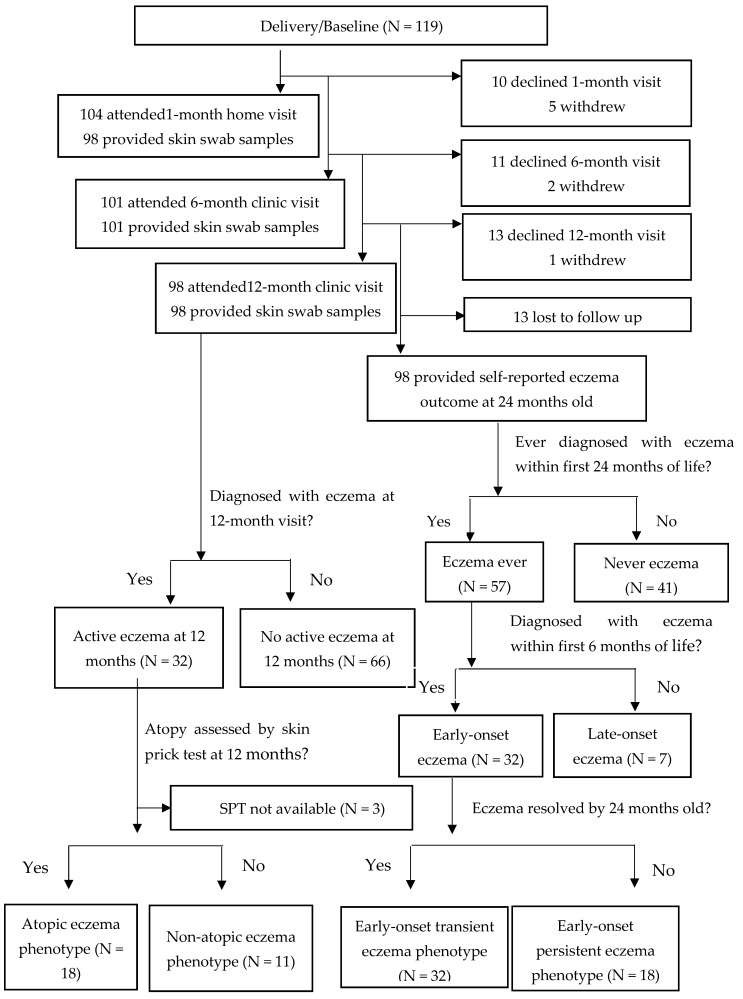
Flow diagram of subjects in this Stool Microbiome and Allergic ReacTion (SMART Baby) study. N:Number.

Between September 2017 and March 2018, 119 Chinese mothers in their late third trimester of pregnancy were randomly approached and asked to sign informed written consent for their babies to participate in the SMART Baby study. Thirty-five percent of the mothers and 29% of the fathers had a history of allergic diseases (Table 1). In total, 104 subjects completed home visits at 1 month, while 101 and 98 subjects attended clinic visits at 6 and 12 months, respectively. When their children were 24 months of age, 98 of the mothers mailed back the self-administered study questionnaire. Skin-swab samples were obtained from 98 of the subjects at 1 month, 101 at 6 months, and 98 at 12 months (Figure 1).

At 6 months, 40 (39.6%) of the subjects had active eczema. By 12 months, 32 (32.7%) of the subjects had been diagnosed with eczema, and by 24 months, 22 (22.4%) reported having eczema. Of the 82 subjects who underwent SPT at 12 months old, 26 (31.7%) were atopic to at least one allergen (Tables S1 and S2). The most common allergens o which the subjects were sensitized were hen’s egg and *D. pteronyssinus*. About two-fifths of the atopic subjects were sensitized to two or more allergens.

### 3.2. Relationship between Atopy and Eczema Persistence

Based on the clinical course of eczema within the first 24 months, we divided children who had eczema at six months of age (i.e., early-onset) into transient and persistent phenotypes (Figure 1). By 24 months, 18 of the children still had active eczema (early-onset persistent phenotype), while the eczema resolved in 32 of the subjects (early-onset transient phenotype). We also divided the subjects with active eczema at 12 months of age into atopic (n = 18) and non-atopic (n = 11) subgroups (Figure 1). The atopic children at 12 months were more likely to have a persistent eczema phenotype (odds ratio (OR) 4.95 and 95% confidence interval (CI) 1.29–19.01). Sensitization to two or more allergens was strongly associated with persistent eczema (OR 25.0, 95% CI 2.70–231.59) (Table S3).

### 3.3. Evolution of Early-Life Skin Microbiome

Following DADA2 quality control, we obtained a total of 6,655,307 sequencing reads from 297 DNA samples collected at the 1-, 6-, and 12-month visits, with a mean number of 22,408 reads per sample. We filtered out the rare reads and clustered a total of 49 ASVs, matching 21 genera in the SILVA v132 99% gene-reference database (Table S4). 

**Figure 2:**
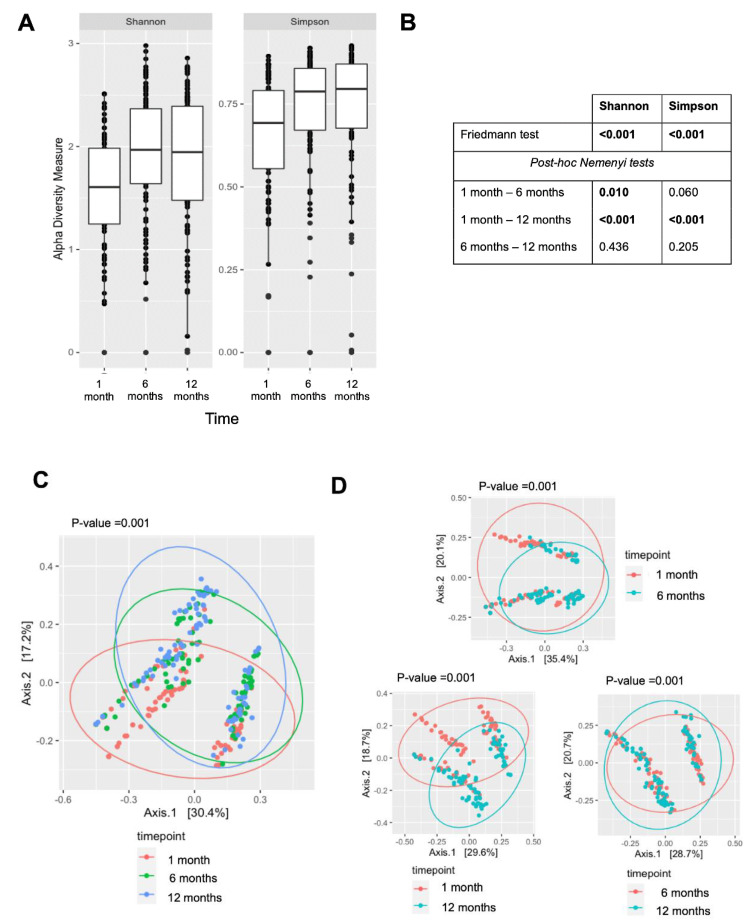
Longitudinal changes in bacterial diversity within the first 12 months of life. (**A**) Boxplots of alpha diversity represented by Shannon and Simpson indices across time. (**B**) Significance of changes in alpha diversity across time was assessed through Friedmann test with post hoc Nemenyi tests. (**C**) Principal coordinates analysis (PCoA) plot based on unweighted UniFrac distance across time. Significance was assessed by the PERMANOVA test. (**D**) Pairwise comparison of beta diversities between sampling points.

**Figure 3:**
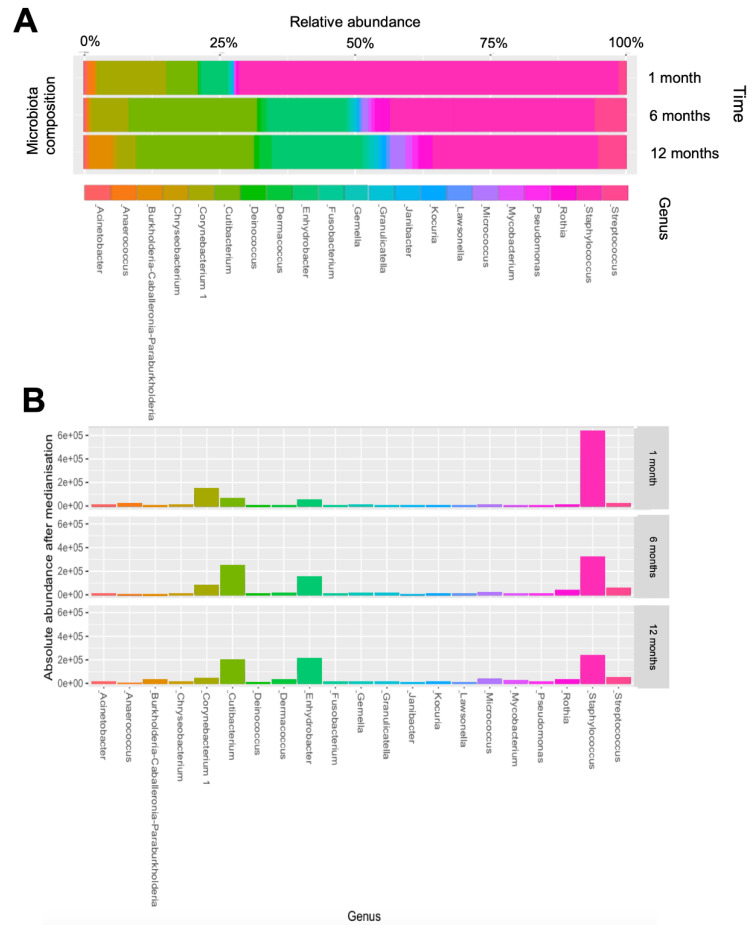
Genus compositions of the skin microbiota from 1 month to 12 months of age. (**A**) A stacked bar chart showing the relative abundances of the 21 filtered genera at each sampling time point. (**B**) Bar charts showing the absolute abundances of the 21 genera at each sampling time point. The number of reads in each microbiome sample was pre-standardized as the median (9730 reads/sample).

There were substantial alterations in the diversity of the skin microbiota over the first year of life (Figure 2). The alpha diversity, represented by the Shannon and Simpson indices, increased during this period (*p* < 0.001, according to the Friedmann test). The post hoc pairwise testing showed a significant difference in the Shannon indices between 1 month and 6 months (*p* < 0.001). These indices remained similar between 6 months and 12 months. The beta diversity, as viewed by the PCoA plot, revealed a substantial shift in the bacterial community structures between the sampling time points (*p* = 0.001 by PERMANOVA). Regarding the skin microbiota composition, the genus *Staphylococcus* was consistently the most prevalent taxa throughout the first year of life (Figure 3), with its relative abundance significantly decreasing from 1 month to 6 months (*p* < 0.001 according to ANCOM-BC; Table S5).

### 3.4. Temporal Effects of Skin-Microbial Diversity on Eczema Phenotypes

**Figure 4:**
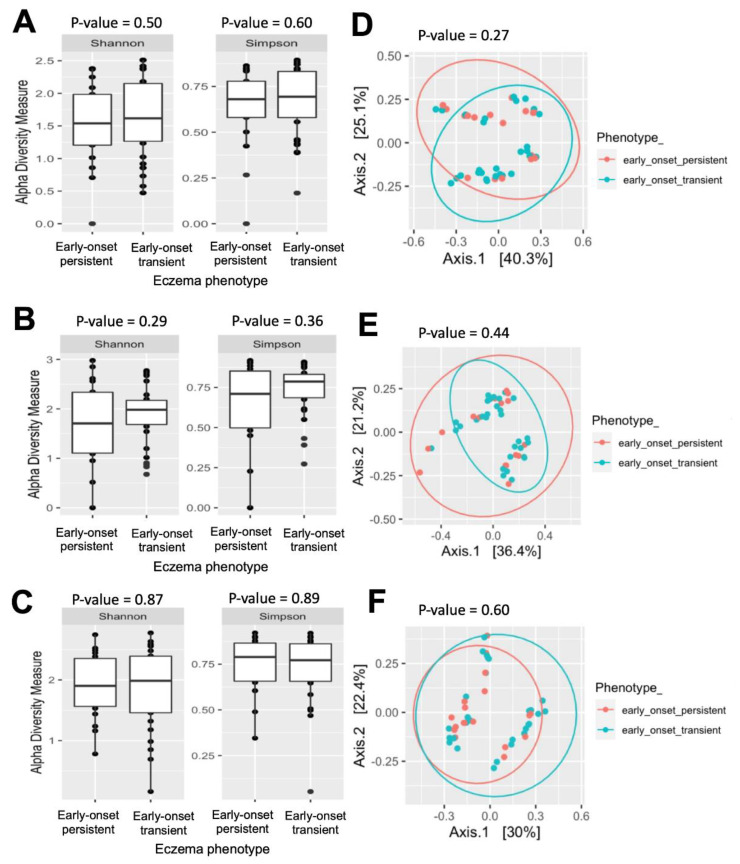
Comparisons of bacterial diversity between transient eczema (N = 32) and persistent eczema (n = 18). Boxplots of alpha diversity represented by Shannon and Simpson indices at (**A**) 1 month, (**B**) 6 months, (**C**) 12 months. Principal coordinates analysis (PCoA) plot of the microbiome samples at (**D**) 1 month, (**E**) 6 months, and (**F**) 12 months based on unweighted UniFrac distance between groups.

Regarding the eczema’s persistence, the Shannon and Simpson indices did not differ between the patients with early-onset transient and persistent eczema phenotypes at any of the sampling times (Figure 4A–C). Furthermore, the bacterial community structures of the patients with the persistent eczema phenotype did not cluster separately from those with the transient phenotype (Figure 4D–F).

**Figure 5:**
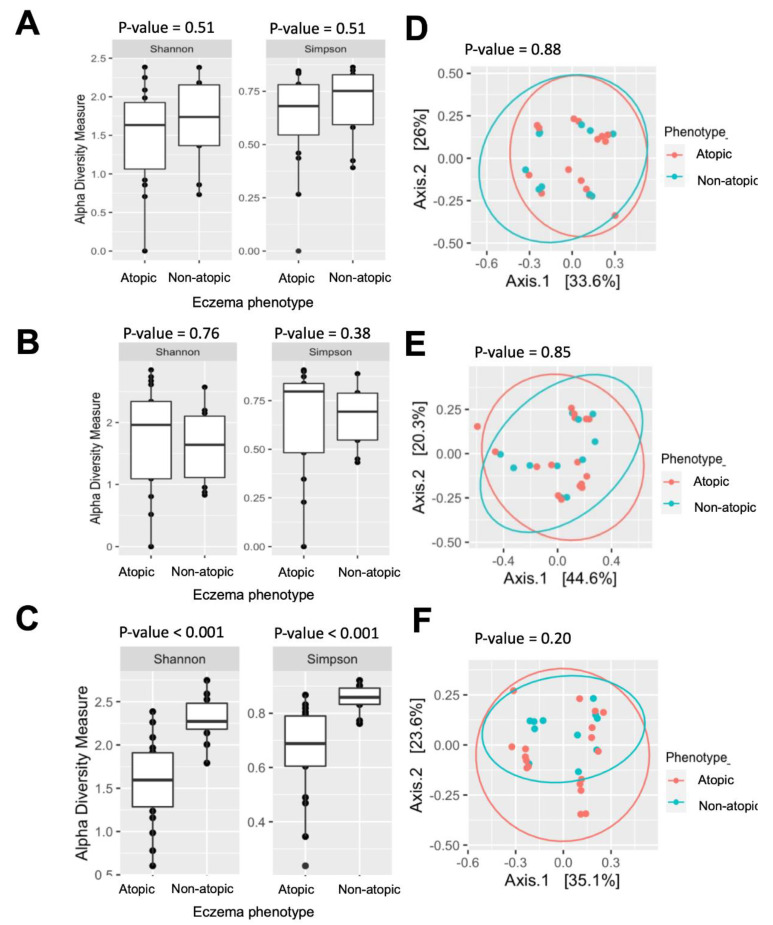
Comparisons of bacterial diversity between atopic eczema (N = 18) and non-atopic eczema (n = 11) at 12 months. Boxplots of alpha diversity represented by Shannon and Simpson indices at (**A**) 1 month, (**B**) 6 months, and (**C**) 12 months. Principal coordinates analysis (PCoA) plot of microbiome samples at (**D**) 1 month, (**E**) 6 months, and (**F**) 12 months based on UniFrac distance between groups.

In terms of atopy status, the patients with the atopic eczema phenotype had a significantly lower alpha diversity at 12 months than those with the non-atopic-eczema phenotype. However, there were no significant differences at earlier time points (Figure 5A–C). Furthermore, the bacterial community structures were similar between the subjects with atopic and non-atopic eczema at all the sampling points (Figure 5D–F).

### 3.5. Skin Microbial Taxa Associated with Eczema Phenotypes

**Figure 6:**
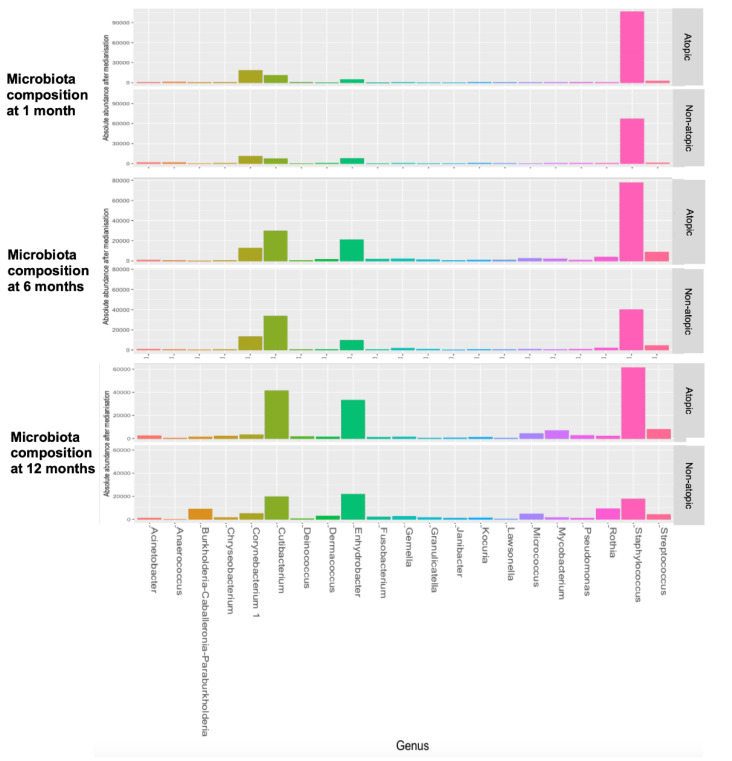
Comparisons of skin-microbiota compositions at the genus level between atopic eczema (n = 18) and non-atopic eczema (n = 11) at 12 months. Bar charts show the absolute abundances of the 21 genera at each sampling time. The number of reads in each microbiome sample was pre-standardized as the median (9730 reads/sample).

The microbiota composition at the genus level was similar between the early-onset transient and persistent eczema phenotypes at all the sampling times (Figure S1). The patients with the atopic eczema phenotype had a higher abundance of the genus *Janibacter* in their skin microbiomes at 6 months of age (*p* < 0.001, Table 2 and Table S6 and Figure 6). However, at the 12-month timepoint, none of the specific genii were found to be different between the children with and without atopy.

## 4. Discussion

Using a birth cohort of Chinese newborns who were unselected for a family history of allergic diseases, we found that the children with atopic-eczema phenotype at 12 months of age had a lower alpha diversity. Specifically, at 6 months, the *Janibacter* appeared to be more abundant on the skins of the children who developed the atopic eczema phenotype when they were 12 months old. Our results suggest that the skin microbiome may play a role in differentiating atopic and non-atopic phenotypes. Lee et al. reported that the skin microbiome might be correlated with cutaneous and systemic immunity, although the underlying mechanisms remained unclear [21]. We also noted that atopic sensitization at 12 months was significantly associated with an increased risk for having persistent eczema by 24 months of age, suggesting that atopy to be a strong risk factor for eczema persistence. 

Interestingly, our results showed that while the skin-microbiome samples from the children with the atopic and non-atopic eczema phenotypes did not form separate clusters in the PCoA plot, the children with the atopic eczema phenotype had a lower alpha diversity in the skin microbiome at 12 months. In this paradox, the children with the atopic eczema phenotype at 12 months old had remarkably fewer skin taxa than those with the non-atopic eczema phenotype. However, the skin taxa of these two groups of children still shared the same branches on the phylogenetic tree, as measured by their unweighted UniFrac distance [22], providing a possible explanation for the discrepancy between the alpha and beta diversity profiles. 

Our results also revealed that the atopic-eczema phenotype had a transiently higher abundance of the genus *Janibacter* at 6 months of age. This suggests that this microbe may have transiently altered cutaneous immunity to upregulate IgE production and initiate eczematous pathogenesis in the skin. While *Janibacter* is one of the most prevalent bacterial taxa on the skin [23], its specific biological functions in human health are yet to be established. The genus *Janibacter* consists of nine different species, which are mainly found in the environment but, rarely, invasive infections by *Janibacter* have been reported in humans [24,25,26], mostly through identification using 16S rRNA gene sequencing. No studies have linked *Janibacter* to skin infections or cutaneous inflammation. It would be important to replicate this finding in other birth-cohort studies and investigate the effect of this bacterium on cutaneous cell lines and skin biopsies from diseased patients in order to delineate its possible roles in eczema pathogenesis.

Two longitudinal Caucasian studies demonstrated that skin-microbial dysbiosis in early life preceded the development of eczema [8,9], but it remains unclear whether skin-microbial profiling may be useful in predicting different eczema phenotypes. Although some studies reported the association between the early-life gut microbiome and eczema development in Hong Kong children [27,28,29], there is limited evidence in this population regarding the skin microbiome and clinical manifestations of childhood eczema. To the best of our knowledge, this study is the first Chinese birth-cohort study to fill in this research gap by investigating the early-life dynamics of the skin microbiome and their potential to predict eczema phenotypes in later childhood.

To evaluate the generalizability of our pilot findings, we compared the baseline characteristics of our birth cohort with the data available on the general population living in Hong Kong SAR. According to the 2022 edition of “Hong Kong Annual Digest of Statistics”, 33.8% of the population aged 15 and over received a post-secondary education in 2021 [30]. In the SMART Baby cohort, this prevalence was higher, which suggested that parents with higher educational levels were more likely to participate in our study. With respect to breastfeeding, the surveys conducted by the Department of health of Hong Kong SAR showed that the exclusive breastfeeding rate at 1 month for babies born in 2018 was 32.6% [31]. In our cohort, the rate of exclusive breastfeeding at 1 month of age was much lower compared with the general population. In terms of eczema prevalence, there were limited local epidemiological data for babies below 2 years of age. However, the International Study of Asthma and Allergies in Childhood (ISSAC) survey in 2015–2016 reported that 30.9% of Hong Kong children aged 6–7 years had experienced eczema at some stage [32]. The prevalence of eczema at 6 months in our cohort was nearly 40%, suggesting that selection bias may have occurred. This could be explained by the finding that mothers with an eczema history were more likely to participate in this birth-cohort study.

This study has several limitations. In terms of the clinical-data collection, we only ascertained eczema diagnosis until 12 months of age by pediatricians. At 24 months, this was based only on parental reporting, because physical attendance at our clinic was impossible due to the COVID-19 pandemic. Eczema occurrence in these older children was recorded by a structured questionnaire modified from our allergy survey, conducted on local pre-school children [11]. Accordingly, this study might have missed more subject data had we not changed the mode of the subject assessment at 24 months. Furthermore, bacterial 16S rRNA gene sequencing, which we used to characterize the skin microbiome, is inferior to whole-genome shotgun sequencing [33]. Thus, our data can only be used to identify skin bacteria down to their genus level. The small sample size in this birth cohort also precluded us from studying the possible association between eczema and individual skin microbes among the young children. Prospective studies with larger sample sizes are needed to determine whether the skin microbiome can predict eczema persistence at 24 months or beyond.

## 5. Conclusions

This birth-cohort study found atopic sensitization to be an important predictor of eczema persistence in toddlers. The skin-microbiome profiles at 6 and 12 months were linked to the atopic eczema phenotype at 12 months old. Future works should explore the relationship and mechanisms between the early-life skin microbiome and the evolution of childhood eczema phenotypes.

## Figures and Tables

**Table 1:** Demographic and clinical characteristics of 119 study participants.

Characteristic	Value
**Maternal characteristics**	
Received education level higher than secondary school, n/N (%)	63/119 (52.9)
History of allergy, n/N (%)	42/119 (35.3)
**Paternal characteristics**	
Received education level higher than secondary school, n/N (%)	60/119 (50.4)
History of allergy, n/N (%)	32/109 (29.4)
**Child characteristics**	
Male, n/N (%)	63/119 (52.9)
Gestational age (weeks)	39.3 ± 1.2 (N = 119)
Birth weight (g)	3145 ± 340 (N = 119)
Born by vaginal delivery, n/N (%)	91/119 (76.5)
Exclusive breastfeeding at 1 month, n/N (%)	26/109 (23.9)
Mixed breast and formula feeding at 1 month, n/N (%)	74/109 (67.9)
Furry pets at home at 1 month (yes), n/N (%)	23/109 (21.1)
Exposure to household smoking at 1 month (yes), n/N (%)	34/109 (31.2)
**Eczema diagnosis, n/N (%)**	
Eczema at 6 months	40/101 (39.6)
Eczema at 12 months	32/98 (32.7)
Eczema at 24 months	22/98 (22.4)
Atopy by skin-prick test at 12 months, n/N (%)	26/82 (31.7)
Sensitization to single tested allergen	15/82 (18.3)
Sensitization to multiple tested allergens	11/82 (13.4)
Received intrapartum antibiotics, n/N(%)	60/119 (50.4)
**Received postnatal antibiotics, n/N (%)**	
Within 1 month after birth	12/109 (11.0)
1 month to 6 months of age	18/100 (18.0)
6 to 12 months of age	22/98 (22.4)
12 to 24 months of age	31/86 (36.0)

Results expressed in number (percentage) or mean ± standard deviation. N: Number.

**Table 2:** The differences in abundance of bacterial genera between atopic eczema (n = 18) and non-atopic eczema (n = 11) at 12 months, tested by ANCOM-BC.

	Non-Atopic Eczema–Atopic Eczema					
Taxa at genus level	Skin Microbiome at 1 Month		Skin Microbiome at 6 Months		Skin Microbiome at 12 Months	
	Beta coefficient	Adjusted *p*-value	Beta coefficient	Adjusted *p*-value	Beta coefficient	Adjusted *p*-value
*Janibacter*	N/A	N/A	−1.085	**<0.001 ^T^**	−0.123	1.000 ^F^

ANCOM-BC, analysis of compositions of microbiomes with bias correction. Bold indicates *p* value < 0.05. ^T^ indicates this genus is differentially abundant. ^F^ indicates this genus is not differentially abundant.

## Supplementary Materials

The following supporting information can be downloaded at: https://www.mdpi.com/article/10.3390/pathogens12050697/s1, Table S1. Profile of allergen sensitization by skin prick test at 12 months; Table S2. Skin prick test profile in 11 subjects with concurrent sensitization to two or more allergens at 12 months; Table S3. Association of clinicodemographic variables with eczema phenotypes stratified by the natural course; Table S4. Taxonomy of 49 filtered amplicon sequencing variants that were present more than three times in at least one-fifth of the samples; Table S5. Bacterial genera that were differentially abundant in our subjects from 1 month to 12 months of age; Table S6. The differentially abundant bacterial genera between atopic eczema (N = 18) and non-atopic eczema (N = 11) at 12 months tested by ANCOM-BC (Full list); Figure S1. Comparisons of skin microbiota compositions at the genus level between early-onset transient eczema (N = 32) and early-onset persistent eczema (N = 18).
