# Supplementary material for: Early-Life Skin Microbial Biomarkers for Eczema Phenotypes in Chinese Toddlers
Source: Pathogens. 2023 May 11;12(5):697. doi: 10.3390/pathogens12050697 (PMC10222142; doi:10.3390/pathogens12050697)
Supplement: Supplementary file 1 [file pathogens-12-00697-s001.zip › pathogens-2320966-supplementary.pdf]

SUPPLEMENTARY MATERIALS

Early-life Skin Microbial Biomarkers for Eczema Phenotypes in Chinese Toddlers

Contents of supplementary materials

Methods

- Characterization of skin microbiome

Results

- **Table S1.** Profile of allergen sensitization by skin prick test at 12 months
- **Table S2.** Skin prick test profile in 11 subjects with concurrent sensitization to two or more allergens at 12 months
- **Table S3.** Association of clinicodemographic variables with eczema phenotypes stratified by the natural course
- **Table S4.** Taxonomy of 49 filtered amplicon sequencing variants that were present more than three times in at least one-fifth of the samples
- **Table S5.** Bacterial genera that were differentially abundant in our subjects from 1 month to 12 months of age
- **Figure S1.** Comparisons of skin microbiota compositions at the genus level between early-onset transient eczema (N=32) and early-onset persistent eczema (N=18).
- **Table S6.** The differentially abundant bacterial genera between atopic eczema (N = 18) and non-atopic eczema (N=11) at 12 months tested by ANCOM-BC (Full list)

Supplementary Methods

Characterization of skin microbiome

Bacterial DNA extraction

All collected skin swab samples were stored at -80ºC until microbial DNA extraction could be carried out in batches. Each batch contained 24 reactions because the centrifuge and hands-free vortex facilities in our lab could handle a maximum of 24 samples at once. In each batch, there were 22 'true' samples and 2 negative controls. The first negative control (blank swab control) included a sterile flock swab and the reagents of the extraction kit, while the second negative control (blank reagent control) included only the reagents. These controls were to be involved in the following library preparation steps and to be sequenced in parallel with DNA samples.

The standard protocol for PureLink™ Microbiome DNA Purification Kit (Invitrogen, USA) was followed exactly for all swab samples. Briefly, each swab was transferred to a bead tube and then incubated with 800 μL of S1 (Lysis buffer) and 100 μL of S2 (Lysis enhancer) at 65ºC for 10 minutes, followed by mechanical beating using a hands-free vortex mixer with horizontal agitation for another 10 minutes. After centrifugation at 14,000 x g for 1 minute, 500 μL of the supernatant was transferred to a clean nuclease-free LoBind tube and mixed with 900 μL of S4 (Binding buffer). The mixture was loaded onto a spin column-tube assembly and centrifuged at 14,000 x g for 1 minute for 2 rounds, with 700 μL of mixture per round. After discarding the flow-through, the spin column was placed in a clean collection tube and loaded with 500 μL of S5 (Wash Buffer), followed by centrifugation at 14,000 x g for 1 minute. To optimize the removal of residual S5 that could interfere with downstream steps, the spin column-tube assembly was further centrifuged at 14,000 x g for 30 seconds. The spin column was placed in a nuclease-free LoBind tube, and 50 μL of S6 (Elution Buffer) was added. The mixture was incubated at room temperature for 1 minute and then centrifuged at 14,000 x g for 1 minute. The flow-through was loaded into the same spin column again and the above elution step was repeated for a second time to slightly increase the DNA yield.

Due to the low-biomass features of skin microbiome samples, additional optimized steps were applied beyond the standard protocol described above. Briefly, for each DNA sample, 5 μL of 3M sodium acetate and 200 μL of cold 100% ethanol (stored at -20ºC) were added and thoroughly mixed before incubating at -20ºC overnight (more than 16 hours). This mixture was later centrifuged at top speed at 4ºC for 30 minutes, and the supernatant was carefully removed. The pellet was washed by adding 500 μL of 75% cold ethanol (stored at -20ºC) and centrifuged at top speed at 4ºC for 10 minutes. The supernatant was removed again, and the washing step was repeated. After removing the supernatant, the pellet was air-dried and reconstituted in 10 μL of S6 for storage and later use.

Library preparation

zExtracted DNA samples were subjected to PCR in batches, with 96 reactions in each batch. To prepare DNA libraries for sequencing, two

rounds of PCR were performed. In the first round of PCR, the 27F/534R primer pair (27F: 5′-AGAGTTTGATCCTGGCTCAG-3′; 534R: 5′ATTACCGCGGCTGCTGG-3′) was used to target and amplify the bacterial V1-V3 hypervariable 16S rRNA gene. Indices and sequencing adapters were attached in the second round of PCR.

The first round of PCR

Each DNA sample underwent the first round of PCR using a 25 μL reaction mixture. The mixture contained 16.875 μL of ddH2O, 2.5 μL of 10X Buffer with MgCl2 (10mM), 1 μL of MgCl2 (25mM), 0.5 μL of dNTP (10mM each), 1 μL of 16SV1V3mix_Forward (5μM), 1 μL of 16SV1V3mix_Reverse (5μM), 0.125 μL of Hot Start plus DNA Taq (5U/μL), and 2 μL of template DNA.

For quality control, each batch (96-well PCR plate) contained two wells of negative controls, one well of mock community control and three wells of technical replicate samples. The negative controls included one well containing 23 μL of reagent, and one containing 23 μL of reagent and 2 μL of ddH2O. The mock community control was 1 μL of ZymoBIOMICS™ Microbial Community DNA Standard (10 ng/μL) and 23 μL of reagent mixture. Technical replicate samples consisted of two within-plate repeats (amplification of one DNA sample repeated in two different wells in the same plate) and one across-plate repeat (amplification of one DNA sample repeated in every plate). The controls above were to be sequenced in parallel with DNA samples.

The PCR conditions consisted of an initial 95°C for 5 min (denaturation), followed by 15 cycles of 94°C for 1 min (denaturation), 55°C for 1 min (annealing), and 72°C for 1 min (elongation), and followed by 15 cycles of 94°C for 1 min (denaturation), 60°C for 1 min (annealing), and 72°C for 1 min (elongation), and a final extension at 72°C for 10 min before storage at 4°C.

The second round of PCR

The resulting PCR products underwent a second round of PCR using a 25 μL reaction mixture. The mixture contained 16.875 μL of ddH2O, 2.5 μL of 10X Buffer with MgCl2 (10mM), 1 μL of MgCl2 (25mM), 0.5 μL of dNTP (10mM each), 1 μL of Nex_Primer P5 (5μM), 1 μL of Nex_Primer P7 (5μM), 1 μL of i5_Golay0396-Golay0301 (stored in 96-well plates) (2.5μM), 0.5 μL of i7_Golay0101 (5uM), 0.125 μL of Hot Start plus DNA Taq (5U/ μL), and 0.5 μL of 1st -round PCR product.

Additionally, two wells were reserved as negative controls: one well containing 24.5 μL of reagent, and one well containing 24.5 μL of reagent and 0.5 μL of ddH2O.

The PCR conditions consisted of an initial 95°C for 5 min (denaturation), followed by 7 cycles of 94°C for 1 min (denaturation), 63°C for 1 min (annealing), and 72°C for 1 min (elongation), and a final extension at 72°C for 10 min before storage at 4°C.

Gel electrophoresis

Successful amplification was verified by 1% agarose gel electrophoresis, which showed an identifiable band at approximately 500 bp.

PCR purification

Successful amplicons were pooled in equimolar quantities (5 μL each). Generally, three 96-well plates of DNA amplicons were pooled into one library, which was then subjected to electrophoresis. The resulting pooled PCR products were excised from agarose gel, purified using QIAquick PCR Purification Kit (Qiagen, Hilden, Germany), and quantified by the Qubit fluorometer before sending out for sequencing.

Bacterial 16S rRNA gene sequencing

Purified libraries were sent to Core Utilities for Cancer Genomics and Pathobiology of Department of Anatomical and Cellular Pathology, Faculty of Medicine, The Chinese University of Hong Kong, for Agilent 2100 Bioanalyzer Service, Qubit Quantification Service, and Illumina MiSeq PE300 sequencing service according to standard protocols. Briefly, for each purified library containing samples from approximately three 96-well plates, the library concentration was first calculated in nM from the Qubit and Bioanalyzer data (mean fragment size of 200-1200bp). The library was then diluted to 4 nM, and 5 μL of the diluted library was denatured by 5 μL 0.2N NaOH for 5 minutes at room temperature. This library was further neutralized by 5 μL 200nM Tris pH 7.0 buffer before dilution to 20 pM with 985 μL HT1 buffer. This library was further diluted to 15 pM with HT1 buffer. To balance the base composition, 150 μL of PhiX control (15 pM) was preloaded in 850 μL of the library (15 pM). Finally, this mixture was loaded with MiSeq v3 kit, and was then placed on the sequencer to run 600 bp PE sequencing.

Table S1. Profile of allergen sensitization by skin prick test at 12 months

Allergen	No. of subjects with allergen sensitization (n=26)	No. of subjects sensitized to one allergen (n=15)	No. of subjects sensitized to multiple allergens (n=11)
Cow's milk	6	2	4
Hen's egg	18	7	11
Soya bean	2	0	2
Peanut	5	0	5
Mixed fish (cod fish, flounder, halibut, mackerel, tuna)	2	0	2
Wheat	2	0	2
Dermatophagoides pteronyssinus	13	6	7

Table S2. Skin prick test profile in 11 subjects with concurrent sensitization to two or more allergens at 12 months

Subject ID	Cow's milk	Hen's egg	Soya bean	Peanut	Mixed fish*	Wheat	*Dermatophagoides pteronyssinus*
Sensitization to 2 allergens							
1		√		√			
2		√					√
3	√	√					
4		√		√			
5		√		√			
6		√					√
Sensitization to 3 allergens							
7	√	√					√
8	√	√					√
Sensitization to 4 allergens							
9		√			√	√	√
Sensitization to 5 allergens							
10		√	√	√		√	√
Sensitization to 6 allergens							
11	√	√	√	√	√		√

* Included cod fish, flounder, halibut, mackerel and tuna.

Table S3. Association of clinicodemographic variables with eczema phenotypes stratified by the natural course

Characteristics	Early-onset transient eczema (N=32)	Early-onset persistent eczema (N=18)	P value
**Maternal characteristics**			
Educational level (higher than secondary school), n/N (%)	16/32 (50.0)	10/18 (55.6)	0.934
History of allergy (yes), n/N (%)	10/32 (31.2)	11/18 (61.1)	0.079
Eczema	8/32 (25.0)	8/18 (44.4)	0.272
Asthma	0/32 (0.0)	2/18 (11.1)	0.241
Allergic rhinitis	8/32 (25.0)	9/18 (50.0)	0.139
Self-reported food allergy	0/32 (0.0)	2/18 (11.1)	0.241
Received intrapartum antibiotics, n/N (%)	18/32 (56.2)	13/18 (72.2)	0.416
**Paternal characteristics**			
Educational level (higher than secondary school), n/N (%)	18/32 (56.3)	8/18 (44.4)	0.612
History of allergy (yes), n/N (%)	10/32 (31.2)	5/18 (27.8)	1.000
Eczema	3/32 (9.4)	2/18 (11.1)	1.000
Asthma	1/32 (3.1)	1/18 (5.6)	1.000
Allergic rhinitis	6/32 (18.8)	2/18 (11.1)	0.760
Self-reported food allergy	0/32 (0.0)	1/18 (5.6)	0.768
**Child characteristics**			
Sex (male), n/N (%)	16/32 (50.0)	9/18 (50.0)	1.000
Gestational age (weeks), mean ± SD	39.2 ± 1.4 (N=32)	39.6 ± 1.2 (N=18)	0.236
Birth weight (g), mean ± SD	3125 ± 354 (N=32)	3189 ± 362 (N=18)	0.551
Mode of delivery (vaginal birth), n/N (%)	23/32 (71.9)	13/18 (72.2)	1.000
Any sibling(s) (yes), n/N (%)	9/32 (28.1)	6/18 (33.3)	0.949
Feeding pattern at 1 month, n/N (%)			0.214
Almost exclusive breastfeeding	6/32 (18.8)	6/18 (33.3)	
Almost exclusive formula feeding	1/32 (3.1)	2/18 (11.1)	
Mixed feeding	25/32 (78.1)	10/18 (55.6)	
Feeding pattern at 6 months			0.165
Almost exclusive breastfeeding	6/31 (19.4)	8/18 (44.4)	
Almost exclusive formula feeding	14/31 (45.2)	5/18 (27.8)	
Mixed feeding	11/31 (35.5)	5/18 (27.8)	
Furry pets at home at 1 month (yes), n/N (%)	4/32 (12.5)	6/18 (33.3)	0.162
Exposure to household smoking at 1 month (yes), n/N (%)	10/32 (31.2)	4/18 (22.2)	0.723
Time of solid food introduction (months), mean ± SD	5.4 ± 0.6 (N=28)	5.5 ± 0.8 (N=15)	0.720
Baseline severity of eczema based on	13/21 (61.9)	14/15 (93.3)	0.079

SCORAD index at 6 months (moderate-to-severe eczema), n/N (%)			
Atopy by Skin Prick Test at 12 months (yes), n/N (%)	8/26 (30.8)	11/16 (68.8)	**0.037**[R]
Atopic sensitisation to two or more tested allergens	1/26 (3.8)	8/16 (50.0)	**<0.001**[S]
Received antibiotics, n/N (%)			
Within 1 month after birth	3/32 (9.4)	2/18 (11.1)	1.000
1 month to 6 months of age	6/30 (20.0)	5/18 (27.8)	0.790
6 to 12 months of age	7/29 (24.1)	4/18 (22.2)	1.000
12 to 24 months of age	10/29 (34.5)	6/18 (33.3)	1.000

SD, standard deviation; *CI*, confidence interval; bold indicates p value < 0.05

[R] the odds ratio between atopy and early-onset persistent eczema is **4.95 (1.29 – 19.01)**

[S] the odds ratio between atopic sensitization to two or more tested allergens and early-onset persistent eczema is **25 (2.7 – 231.59)**

Table S4. Taxonomy of 49 filtered amplicon sequencing variants that were present more than three times in at least one-fifth of the samples

ASV ID	Kingdom	Phylum	Class	Order	Family	Genus	Species	Confidence	Aggregated genus
04ada4e14d99e14da4943db86b935051	Bacteria	Proteobacteria	Gammaproteobacteria	Pseudomonadales	Moraxellaceae	Acinetobacter	Acinetobacter ursingii NIPH 706	0.8468753	Acinetobacter
19efac48457c44e44de9dee0a284c57e	Bacteria	Proteobacteria	Gammaproteobacteria	Pseudomonadales	Moraxellaceae	Acinetobacter		0.9999988	
c988a0fb08d7ba6b38ee5b64ccdd1a02	Bacteria	Firmicutes	Clostridia	Clostridiales	Family XI	Anaerococcus		1.0000000	Anaerococcus
2f7571ef1bad4e37cc1b4b0c2fd7ee2a	Bacteria	Firmicutes	Clostridia	Clostridiales	Family XI	Anaerococcus	uncultured bacterium	0.7542889	
d498b348421fbeb868e78fd72bf897bd	Bacteria	Proteobacteria	Gammaproteobacteria	Betaproteobacteriales	Burkholderiaceae	Burkholderia-Caballeronia-Paraburkholderia		1.0000000	Burkholderia-Caballeronia-Paraburkholderia
3b1573b901cccd4c73b650115173c779	Bacteria	Bacteroidetes	Bacteroidia	Flavobacteriales	Weeksellaceae	Chryseobacterium	Chryseobacterium hominis	0.9706997	Chryseobacterium
a37b59845d587f24cab7f5f053465d71	Bacteria	Actinobacteria	Actinobacteria	Corynebacteriales	Corynebacteriaceae	Corynebacterium 1	Corynebacterium pseudodiphtheriticum	0.9983807	Corynebacterium 1
04ae6d4e93605dc0fd87868b6069bbbf	Bacteria	Actinobacteria	Actinobacteria	Corynebacteriales	Corynebacteriaceae	Corynebacterium 1	Corynebacterium pseudogenitalium ATCC 33035	0.7531123	

727bdda2bfd37e7ea0e3968480ee22f5	Bacteria	Actinobacteria	Actinobacteria	Propionibacteriales	Propionibacteriaceae	Cutibacterium	[Propionibacterium] humerusii P08	0.9076308	
2c38d9d5742202da694d4721608fd637	Bacteria	Actinobacteria	Actinobacteria	Propionibacteriales	Propionibacteriaceae	Cutibacterium		1.0000000	Cutibacterium
856925f916eba95f669698c572f88d93	Bacteria	Actinobacteria	Actinobacteria	Propionibacteriales	Propionibacteriaceae	Cutibacterium	Propionibacterium sp. KPL1844	0.9976461	
698c357ed6176262d12440ccd4abb093	Bacteria	Deinococcus-Thermus	Deinococci	Deinococcales	Deinococcaceae	Deinococcus	Deinococcus antarcticus	0.9999999	Deinococcus
8c78f224069832cd7fd7e9efcf616ab9	Bacteria	Actinobacteria	Actinobacteria	Micrococcales	Dermacoccaceae	Dermacoccus	Dermacoccus sp. PE3	0.9991661	Dermacoccus
8a8681bd58fa527cb40dc43c44a5f7cc	Bacteria	Actinobacteria	Actinobacteria	Micrococcales	Dermacoccaceae	Dermacoccus		0.9999998	
5325c97ed1a497a5a586815b59aed735	Bacteria	Proteobacteria	Gammaproteobacteria	Pseudomonadales	Moraxellaceae	Enhydrobacter		0.9982389	
12ba80af9b705ca0875590498ffa2a76	Bacteria	Proteobacteria	Gammaproteobacteria	Pseudomonadales	Moraxellaceae	Enhydrobacter	uncultured bacterium	0.9633295	Enhydrobacter
0dc806f76606ad108e0ad08c8beab95c	Bacteria	Proteobacteria	Gammaproteobacteria	Pseudomonadales	Moraxellaceae	Enhydrobacter	uncultured bacterium	0.7971678	
3a6294eb3d425bab566e18	Bacteria	Proteobacteria	Gammaproteobact	Pseudomonadales	Moraxellaceae	Enhydrobacter	uncultured bacterium	0.9297902	

0ea33b5d1c			eria						
1cbda894755 e11f49abb454 73b87fa68	Bacteri a	Proteoba cteria	Gammap roteobact eria	Pseudomo nadales	Moraxell aceae	Enhydrobact er	uncultured bacterium	0.89769 80	
d5aefd28a1cf 55f766255eb8 f137bb12	Bacteri a	Proteoba cteria	Gammap roteobact eria	Pseudomo nadales	Moraxell aceae	Enhydrobact er	uncultured bacterium	0.95114 23	
bb3ec7a794b 0a019a15b1a 0cca544d7d	Bacteri a	Proteoba cteria	Gammap roteobact eria	Pseudomo nadales	Moraxell aceae	Enhydrobact er	uncultured bacterium	0.88966 49	
71c05ef4f89bf da417762c6a 3a199da5	Bacteri a	Fusobact eria	Fusobact eriia	Fusobacte riales	Fusobact eriaceae	Fusobacteriu m		1.00000 00	Fusobacterium
4704b0a904f3 d62f6496b0d c9ce2fb93	Bacteri a	Firmicut es	Bacilli	Bacillales	Family XI	Gemella	uncultured bacterium	0.80921 56	Gemella
46ec4ed0a75 b0fcf231f1e4f f262fa8d	Bacteri a	Firmicut es	Bacilli	Lactobacill ales	Carnoba cteriacea e	Granulicatell a	uncultured bacterium	0.75103 40	Granulicatella
0db1ad2d6b4 b45fa3e3e40a a9f5ee692	Bacteri a	Actinoba cteria	Actinoba cteria	Micrococc ales	Intraspor angiacea e	Janibacter	Janibacter anophelis	0.94137 54	Janibacter
416480b43f1a e4a764980f50 b7535604	Bacteri a	Actinoba cteria	Actinoba cteria	Micrococc ales	Micrococ caceae	Kocuria		0.99999 99	Kocuria
51afe0f294ef4 1a2de597e8f0 135596b	Bacteri a	Actinoba cteria	Actinoba cteria	Micrococc ales	Micrococ caceae	Kocuria	Kocuria palustris	0.73868 22	
0df52bde0ac2	Bacteri	Actinoba	Actinoba	Corynebac	Coryneb	Lawsonella	Lepisosteus	0.92105	Lawsonella

87bb20a6c5a9a66201f2	a	cteria	cteria	teriales	acteriaceae		oculatus (spotted gar)	42	
578ebb5c9151678a0c2a6cd159f52a74	Bacteria	Actinobacteria	Actinobacteria	Micrococcales	Micrococcaceae	Micrococcus	Micrococcus luteus	0.7950362	Micrococcus
4de6b153aa2a3a8c8c78a63b0a234807	Bacteria	Actinobacteria	Actinobacteria	Micrococcales	Micrococcaceae	Micrococcus	Micrococcus luteus	0.7598114	
46ce74203d097a825237adc200b8c4fb	Bacteria	Actinobacteria	Actinobacteria	Corynebacteriales	Mycobacteriaceae	Mycobacterium	Mycobacterium sp. Myc399	0.9713264	Mycobacterium
6d2b3c2c508427b0d7dde5e9d8e7ff2e	Bacteria	Proteobacteria	Gammaproteobacteria	Pseudomonadales	Pseudomonadaceae	Pseudomonas		0.9925990	Pseudomonas
528e92fe752fa87f855f5536b33979d1	Bacteria	Proteobacteria	Gammaproteobacteria	Pseudomonadales	Pseudomonadaceae	Pseudomonas	Pseudomonas psychrotolerans L19	0.9964812	
abfbcd31ee01d082d222b8afc5ecd572	Bacteria	Actinobacteria	Actinobacteria	Micrococcales	Micrococcaceae	Rothia	Rothia mucilaginosa M508	0.7768646	Rothia
443fb2d16fc7d839c24c5a7a7826dec6	Bacteria	Actinobacteria	Actinobacteria	Micrococcales	Micrococcaceae	Rothia		1.0000000	
0a6e5f1f3e123ed71ecd266e6f15bae1	Bacteria	Actinobacteria	Actinobacteria	Micrococcales	Micrococcaceae	Rothia		0.9998781	
46d179efc5684cf91a6cac9ae3743529	Bacteria	Firmicutes	Bacilli	Bacillales	Staphylococcaceae	Staphylococcus		0.9999997	Staphylococcus

20860b730ce d34c41cb21fc 41c9f02b2	Bacteri a	Firmicut es	Bacilli	Bacillales	Staphylo coccacea e	Staphylococc us		0.99999 99	
483181f68a19 97d8d691fc99 eb4d8919	Bacteri a	Firmicut es	Bacilli	Bacillales	Staphylo coccacea e	Staphylococc us	Staphylococcus aureus	0.98268 46	
28791a195fb1 6e432f328fc3 8c217a16	Bacteri a	Firmicut es	Bacilli	Bacillales	Staphylo coccacea e	Staphylococc us		0.99702 69	
b2339ee5c951 8a0b95176da 819e0ccc5	Bacteri a	Firmicut es	Bacilli	Bacillales	Staphylo coccacea e	Staphylococc us		0.99686 71	
de3433571b8 70a3b3f49bfe 307718094	Bacteri a	Firmicut es	Bacilli	Bacillales	Staphylo coccacea e	Staphylococc us		0.99999 99	
bbd9195eb17 5dbfe0bbe51 314886d77c	Bacteri a	Firmicut es	Bacilli	Bacillales	Staphylo coccacea e	Staphylococc us	Staphylococcus warneri	0.73051 68	
1e002095b1f9 5aa73df22cf5 3653519e	Bacteri a	Firmicut es	Bacilli	Bacillales	Staphylo coccacea e	Staphylococc us		0.99730 17	
c4f81b616f1c 91f7c2cb1180 597aaeaa	Bacteri a	Firmicut es	Bacilli	Bacillales	Staphylo coccacea e	Staphylococc us		0.99999 95	
adb56b97299 ab7fb87729cc b2778908a	Bacteri a	Firmicut es	Bacilli	Bacillales	Staphylo coccacea e	Staphylococc us		0.99736 12	
b7a385c563b 38ce7d2097af	Bacteri a	Firmicut es	Bacilli	Bacillales	Staphylo coccacea	Staphylococc us		0.99692 32	

da10905b0					e				
dc4c06e76c80792a6dd6a5170e96a392	Bacteria	Firmicutes	Bacilli	Lactobacillales	Streptococcaceae	Streptococcus	Streptococcus salivarius subsp. thermophilus	0.8207073	Streptococcus
453e5a05d5ed98456ca12b32e72f4ab9	Bacteria	Firmicutes	Bacilli	Lactobacillales	Streptococcaceae	Streptococcus	Streptococcus pneumoniae	0.8031018	

Table S5. Bacterial genera that were differentially abundant in our subjects from 1 month to 12 months of age

Taxa at genus level	6 months - 1 month		12 months - 6 months		12 months - 1 month	
	β	P_{adj}	β	P_{adj}	β	P_{adj}
Corynebacterium 1	−1.847	**<0.001^T**	−1.452	**<0.001^T**	−3.413	**<0.001^T**
Lawsonella	0.300	1.000^F	−0.006	1.000^F	0.180	1.000^F
Mycobacterium	−0.163	1.000^F	0.737	0.930^F	0.461	1.000^F
Dermacoccus	0.770	0.265^F	0.638	1.000^F	1.295	**0.003^T**
Janibacter	−0.423	1.000^F	0.589	0.803^F	0.052	1.000^F
Kocuria	0.163	1.000^F	0.631	0.930^F	0.680	0.268^F
Micrococcus	0.780	0.282^F	0.012	1.000^F	0.678	0.619^F
Rothia	1.548	**<0.001^T**	−0.030	1.000^F	1.404	**0.001^T**
Cutibacterium	1.155	**0.001^T**	−0.981	**0.019^T**	0.061	1.000^F
Chryseobacterium	−0.109	1.000^F	0.061	1.000^F	−0.162	1.000^F
Deinococcus	0.165	1.000^F	0.899	0.211^F	0.950	**0.033^T**
Gemella	0.337	1.000^F	−0.216	1.000^F	0.008	1.000^F
Staphylococcus	−2.136	**<0.001^T**	−1.037	0.082^F	−3.287	**<0.001^T**
Granulicatella	1.319	**0.001^T**	0.338	1.000^F	1.542	**<0.001^T**
Streptococcus	1.088	0.203^F	0.085	1.000^F	1.059	0.148^F
Anaerococcus	−2.518	**<0.001^T**	−0.179	1.000^F	−2.810	**<0.001^T**
Fusobacterium	0.826	0.070^F	0.953	0.187^F	1.666	**<0.001^T**
Burkholderia-Caballeronia-Paraburkholderia	−1.249	**<0.001^T**	2.604	**<0.001^T**	1.240	**0.023^T**
Acinetobacter	−0.191	1.000^F	0.037	1.000^F	−0.268	1.000^F
Enhydrobacter	1.046	0.062^F	−0.127	1.000^F	0.806	0.268^F
Pseudomonas	0.080	1.000^F	−0.409	1.000^F	−0.444	1.000^F

ANCOM-BC, analysis of compositions of microbiomes with bias correction.
Analyzed by ANCOM-BC, with bold results indicating adjusted *p*-value < 0.05.
^{T} indicates this genus was differentially abundant.

F indicates this genus was not differentially abundant.

Figure S1. Comparisons of skin microbiota compositions at the genus level between early-onset transient eczema (N=32) and early-onset persistent eczema (N=18). Bar charts show the absolute abundances of the 21 genera at each sampling time. The number of reads in each microbiome sample was pre-standardized as the median (9730 reads/sample)

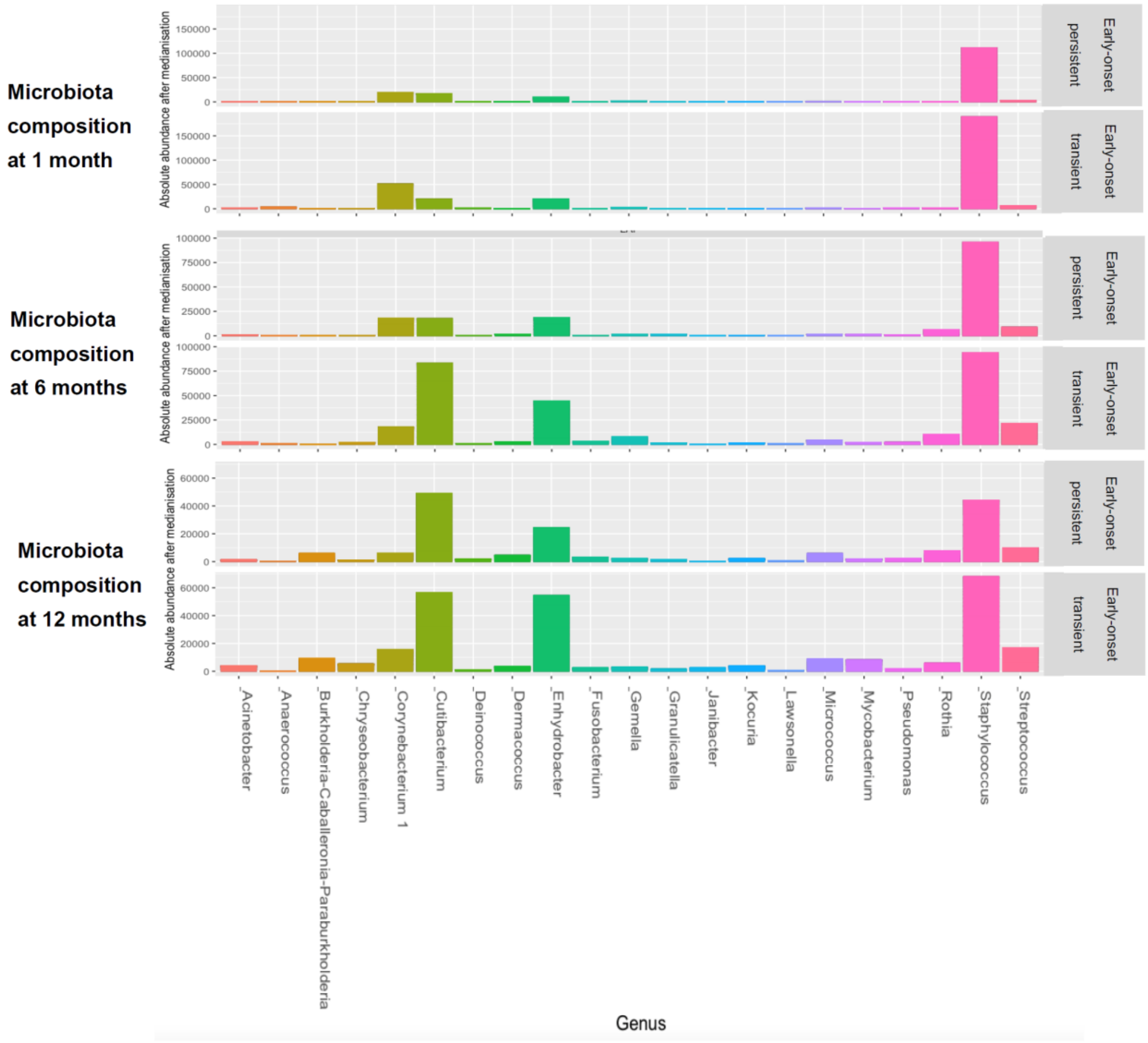

Table S6. The differentially abundant bacterial genera between atopic eczema (N=18) and non-atopic eczema (N=11) at 12 months as tested by ANCOM-BC (Full list)

	**Non-atopic eczema – Atopic eczema**					
	Skin microbiome at 1 month		Skin microbiome at 6 months		Skin microbiome at 12 months	
Taxa at genus level	Beta coefficients	Adjusted p-values	Beta coefficients	Adjusted p-values	Beta coefficients	Adjusted p-values
Corynebacterium 1	0.236	1.000[F]	0.403	1.000[F]	1.773	0.143[F]
Lawsonella	−0.514	1.000[F]	0.324	1.000[F]	−0.525	1.000[F]
Mycobacterium	0.426	1.000[F]	−0.133	1.000[F]	−0.139	1.000[F]
Dermacoccus	0.972	1.000[F]	−1.099	1.000[F]	0.570	1.000[F]
Janibacter	N/A	N/A	−1.085	**<0.001[T]**	−0.123	1.000[F]
Kocuria	0.138	1.000[F]	−0.132	1.000[F]	1.083	1.000[F]
Micrococcus	−0.766	1.000[F]	−0.859	1.000[F]	1.887	0.628[F]
Rothia	−0.060	1.000[F]	−0.559	1.000[F]	1.372	0.780[F]
Cutibacterium	0.341	1.000[F]	0.325	1.000[F]	0.209	1.000[F]
Chryseobacterium	0.699	1.000[F]	0.506	1.000[F]	−0.231	1.000[F]
Deinococcus	−0.836	1.000[F]	0.389	1.000[F]	1.294	1.000[F]
Gemella	−0.067	1.000[F]	−0.602	1.000[F]	1.046	1.000[F]
Staphylococcus	−0.010	1.000[F]	0.013	1.000[F]	−0.523	1.000[F]

Granulicatella	N/A	N/A	2.098	0.747[F]	1.217	1.000[F]
Streptococcus	−0.661	1.000[F]	0.230	1.000[F]	−0.552	1.000[F]
Anaerococcus	−0.405	1.000[F]	−0.106	1.000[F]	N/A	N/A
Fusobacterium	N/A	N/A	−0.110	1.000[F]	0.483	1.000[F]
Burkholderia-Caballeronia-Paraburkholderia	−0.734	1.000[F]	−0.041	1.000[F]	1.527	1.000[F]
Acinetobacter	1.188	1.000[F]	−0.142	1.000[F]	−0.386	1.000[F]
Enhydrobacter	0.548	1.000[F]	−0.162	1.000[F]	0.158	1.000[F]
Pseudomonas	−0.044	1.000[F]	0.919	1.000[F]	−1.021	1.000[F]
